# Ultra‐Large Spontaneous Portosystemic Shunt is Correlated With a Higher Hepatic Venous Pressure Gradient and Increased Risk of Hepatic Encephalopathy

**DOI:** 10.1155/cjgh/4788170

**Published:** 2026-03-16

**Authors:** Xiao-juan Lei, Yu-bing Jiao, Xin-hui Huang, Sheng-zhao Li, Qiao Ke, Wu-hua Guo

**Affiliations:** ^1^ Department of Interventional Radiology, Mengchao Hepatobiliary Hospital of Fujian Medical University, Fuzhou, Fujian, 350025, China, fjmu.edu.cn; ^2^ Department of Hepatopancreatobiliary Surgery, Zhejiang Cancer Hospital, Hangzhou Institute of Medicine (HIM), Chinese Academy of Sciences, Hangzhou, Zhejiang, 310022, China, cas.cn

**Keywords:** cirrhosis, hepatic encephalopathy, hepatic venous pressure gradient, liver function, portal hypertension, spontaneous portosystemic shunts

## Abstract

**Background:**

Spontaneous portosystemic shunts (SPSSs) frequently develop in individuals with cirrhosis. However, their clinical impact on the hepatic venous pressure gradient (HVPG) and liver function remains complex and a subject of ongoing debate. This retrospective study evaluated the relationship between SPSS diameter, particularly ultra‐large SPSS, and HVPG, liver dysfunction, and their predictive value in patients with cirrhosis.

**Methods:**

A retrospective study was conducted on 90 cirrhotic patients. The patients were categorized into three groups: no SPSS (none or *φ* < 3 mm), small SPSS (3 ≤ *φ* < 8 mm), and large SPSS (*φ* ≥ 8 mm). The large SPSS group was further stratified into large (8 ≤ *φ* < 14.5 mm) and ultra‐large (*φ* ≥ 14.5 mm) subgroups based on a receiver operating characteristic (ROC)−derived cutoff for hepatic encephalopathy (HE). Clinical parameters, HVPG, and vascular diameters were analyzed.

**Results:**

The large SPSS group had higher total bilirubin and a higher incidence of HE than the no SPSS group. Within the large SPSS cohort, the ultra‐large subgroup (≥ 14.5 mm) demonstrated significantly worse liver function (lower albumin, higher bilirubin, prolonged PT, increased INR, and poorer Child–Pugh/MELD scores), a higher proportion of Child–Pugh Class C, and elevated HVPG values (18.7 vs. 16.6 mmHg, *p* = 0.03) compared to the large subgroup. Critically, SPSS diameter showed a significant positive correlation with HVPG (*R* = 0.314, *p* = 0.013). The incidence of HE was also significantly higher in the ultra‐large subgroup (55.52% vs. 11%, *p* = 0.001).

**Conclusions:**

The portal pressure‐lowering effect of SPSS does not correlate linearly with its diameter, exhibiting a threshold‐dependent attenuation as shunt size exceeds 14.5 mm. This cutoff may serve as a predictor of increased risk for HE and liver dysfunction in cirrhosis, supporting its potential role in clinical risk stratification and decision‐making for cirrhosis patients with SPSS.

## 1. Introduction

Cirrhosis is an end‐stage liver disease resulting from chronic hepatic injury. It is characterized by hepatic hypoplasia, progressive liver dysfunction, and portal hypertension, all of which can lead to serious or potentially life‐threatening complications [1, 2]. A key feature of portal hypertension is the formation of portal collateral circulation, including spontaneous portosystemic shunts (SPSSs), abnormal vascular connections that divert portal blood flow directly into the systemic circulation, thereby bypassing the liver [3, 4]. Although SPSS may reduce portal pressure and help prevent complications such as variceal bleeding, they also exacerbate hepatic encephalopathy (HE) by allowing systemic accumulation of toxins (e.g., ammonia), promote esophagogastric variceal rupture and hemorrhage, and contribute to other adverse outcomes. These effects can accelerate liver dysfunction, lead to refractory HE, and ultimately increase mortality in patients with cirrhosis [5–7]. The clinical impact of SPSS in cirrhosis remains complex and controversial, with evidence suggesting both beneficial and harmful consequences. This lack of consensus highlights the need for further investigation into the clinical significance of SPSS and its potential antihypertensive effects [8, 9].

The hepatic venous pressure gradient (HVPG) is the gold standard for assessing the severity of portal hypertension [10]. It indirectly reflects portal venous pressure and is widely used to evaluate disease progression and monitor treatment outcomes [11, 12]. A normal HVPG value ranges from 3 to 5 mm Hg, whereas portal hypertension is diagnosed when HVPG is ≥ 10 mm Hg. An HVPG of ≥ 12 mm Hg increases the risk of complications, such as variceal bleeding and decompensation [3, 10, 13, 14]. Given that SPSS arises from hemodynamic changes in the portal vein due to sustained hypertension, it is crucial to assess the correlation between HVPG and SPSS diameter. Recent studies have demonstrated that as the SPSS diameter increases, patients often experience worse liver function and a significantly higher incidence of complication [15, 16]. Most studies categorize a large SPSS as one with a maximum diameter of ≥ 8 mm or when any of multiple shunt diameters meet this criterion [7, 17]. However, our clinical observations indicate that large SPSS can range widely from 8 mm to over 30 mm in diameter, complicating the assessment of their impact when grouped or pooled together. As such, it is necessary to investigate the correlation between HVPG and SPSS diameters by further stratifying large SPSS into ultra‐large diameters to achieve a more accurate evaluation.

We conducted this study to better understand the effects of different SPSS diameter ranges on the prognosis of cirrhosis patients. The findings from the present study may provide a detailed analysis based on HVPG measurements.

## 2. Materials and Methods

### 2.1. Patients

All procedures in this study conformed to the ethical standards of the institutional and national committees on human experimentation and complied with the Declaration of Helsinki (1975), as revised in 2008 (5). Informed consent was obtained from all patients before being included in the study. The study protocol was approved by the Ethics Committees of Mengchao Hepatobiliary Hospital of Fujian Medical University (Approval number: 2024_109_01).

A retrospective analysis was conducted on cirrhotic patients aged 30–75 years who underwent HVPG measurement between April 2018 and July 2025. The diagnosis of cirrhosis was confirmed by either liver biopsy or imaging findings.

The exclusion criteria were as follows:

1. Patients with tumors.
2. Patients who had undergone splenectomy.
3. Patients with hepatic artery−portal vein fistula.
4. Patients who had received embolization.
5. Patients who had undergone transjugular intrahepatic portosystemic shunt (TIPS).
6. Patients with HVPG values < 10 mmHg.
7. Patients diagnosed with noncirrhotic liver disease based on liver biopsy.

### 2.2. HVPG Measurement

HVPG measurement was carried out using imaging equipment, including Toshiba INFINIX‐9000C DSA, PHILIPS Azurion7M20 DSA, PHILIPS Brilliance iCT, and Neusoft NeuViz Epoch CT. HVPG was measured following interventional pressure measurement methods as reported previously [18]. In brief, a Fogarty 6F balloon catheter (Edwards, USA) was employed via the right jugular vein approach, with the catheter tip connected to a pressure transducer. Subsequent steps involved an initial zero‐point measurement with open sensors prior to catheter placement, followed by measuring free hepatic venous pressure (FHVP) and hepatic vein wedge pressure (WHVP). Each measurement was repeated three times, and the average values were recorded to ensure accuracy. The HVPG was calculated using the formula: HVPG = WHVP − FHVP. The transjugular HVPG measurements and interpretations were carried out by three physicians with 20, 10, and 7 years of experience in interventional radiology.

### 2.3. Clinicopathological Parameters

Clinicopathological parameters were collected and analyzed, including the etiology of cirrhosis, cirrhosis‐related complications (e.g., ascites, HE, and ruptured esophagogastric varices), total bilirubin, albumin, prothrombin time (PT), international normalized ratio (INR), Child–Pugh score and classification, the Model for End‐stage Liver Disease (MELD), and MELD‐Na scores.

### 2.4. Radiological Assessments

Two examiners independently reviewed contrast‐enhanced abdominal CT or MRI scans to assess SPSS by measuring the diameters of the shunts, main portal vein (MPV), left and right portal veins (LPV and RPV), superior mesenteric vein (SMV), and splenic vein (SV). At a point approximately 1‐2 cm from the origin of the portal vein branch, a straight segment of the lumen was selected, and the internal diameter was measured using electronic calipers perpendicular to the long axis of the vessel, as well as the presence of portal vein thrombosis (PVT) was evaluated. Liver and spleen volumes were measured using the Philips Nebula Workstation (Philips IntelliSpace Portal ISP V6), selecting the portal venous phase for analysis. The software automatically delineated the liver contour based on a predefined threshold. Manual adjustments were applied to exclude nonparenchymal structures, such as the gallbladder, bile ducts, blood vessels, fatty deposits, and adjacent tissues in the hepatic fissures. Spleen volume was determined using a smart region‐of‐interest tool that identified the splenic parenchyma, after which the software calculated the respective organ volumes.

### 2.5. Statistical Analysis

Descriptive statistics were utilized to summarize the data, including means, medians, standard deviations, ranges, and quartiles for continuous variables, as well as frequency tables and bar charts for categorical variables. Normality tests were conducted to assess data distribution. For continuous variables with a normal distribution, group comparisons were performed using the independent‐samples *t*‐test (e.g., for comparisons between two subgroups) or one‐way ANOVA (e.g., for comparisons among three groups), whereas the Mann−Whitney *U* test or Kruskal−Wallis H test was utilized for non‐normally distributed data. The chi‐square test and Fisher’s exact test were employed for categorical variables. Predicted probabilities were calculated using the Hosmer−Lemeshow test in a binary logistic regression. Receiver operating characteristic (ROC) curves were used for joint diagnostic analysis, and sensitivity, specificity, and the Youden Index were calculated to determine the optimal cutoff value. Pearson’s correlation was applied for normally distributed continuous variables and Spearman’s rank correlation for non‐normally distributed variables. All analyses were performed using IBM SPSS Statistics Version 25.0.

## 3. Results

### 3.1. Patient Characteristics

A total of 90 patients with cirrhotic portal hypertension who underwent HVPG measurement were retrospectively enrolled in the study. These patients were categorized into three groups based on the status of SPSS and the diameter of the shunt tract (*φ*) as follows: No SPSS (none or *φ* < 3 mm), small SPSS (3 mm ≤ *φ* < 8 mm), and large SPSS (*φ* ≥ 8 mm). The baseline characteristics of the three groups are summarized in Table 1. Analysis revealed no significant differences among the groups in terms of gender, age, etiology, albumin, total bilirubin, PT, INR, Child–Pugh, MELD, MELD‐Na, and HVPG (*p* > 0.05). The large SPSS group exhibited elevated total bilirubin levels relative to the other two groups (32.4 vs. 24.1 vs. 21.7; *p* = 0.04).

**TABLE 1:** Baseline characteristics of the patients stratified by SPSS diameter.

	Total (*n* = 90)	No SPSS (*n* = 20)	Small SPSS (*n* = 20)	Large SPSS (*n* = 50)	*p* value
Gender					0.495
Female	23 (25.6%)	3 (15%)	6 (30%)	14 (28%)	
Male	67 (74.4%)	17 (85%)	14 (70%)	36 (72%)	
Age (years)	53.4 ± 11.6	52.9 ± 10.6	51.9 ± 14.9	54.2 ± 10.6	0.766
Etiology					0.943
HBV	61 (67.8%)	13 (65%)	14 (70%)	34 (68%)	
Others	29 (32.2%)	7 (35%)	6 (30%)	16 (32%)	
Albumin (g/L)	32.5 ± 5.6	33.6 ± 4.7	34.1 ± 5.2	31.5 ± 5.9	0.125
Total bilirubin (μmol/L)	25.6 (19.3–41.5)	21.7 (16.7–31.4)	24.1 (15.7–32.3)	32.4 (21.5–50.8)	0.040
PT (s)	16.1 (15–17.7)	16 (15–17.2)	15.5 (14.7–17.2)	16.7 (15.3–18.6)	0.121
INR	1.3 (1.17–1.46)	1.27 (1.18–1.39)	1.23 (1.13–1.41)	1.34 (1.2–1.56)	0.126
Child–Pugh	7.5 (6–9)	7 (5–9.5)	6.5 (6–8)	8 (6–9.25)	0.092
Child–Pugh grade					0.273
A	32 (35.6%)	9 (45%)	10 (50%)	13 (26%)	
B	38 (42.2%)	6 (30%)	7 (35%)	25 (50%)	
C	20 (22.2%)	5 (25%)	3 (15%)	12 (24%)	
MELD	11 (9.8–14)	10.5 (9–15.5)	10.5 (9–11.8)	12.5 (10–15.3)	0.052
MELD‐Na	11 (9–14)	11 (9–15.9)	11 (9.3–12.8)	11 (9–14.1)	0.792
HVPG values	17.8 (15–20)	17 (15–20.8)	17 (15–20)	20 (16–22)	0.223

*Note:* MELD, Model for End‐stage Liver Disease.

Abbreviations: EGVB, esophageal‐gastric variceal bleeding; HBV, hepatitis B virus; HVPG, hepatic venous pressure gradient; INR, international normalized ratio; PT, prothrombin time.

### 3.2. Portal Venous System Imaging Measurements

**FIGURE 1:**
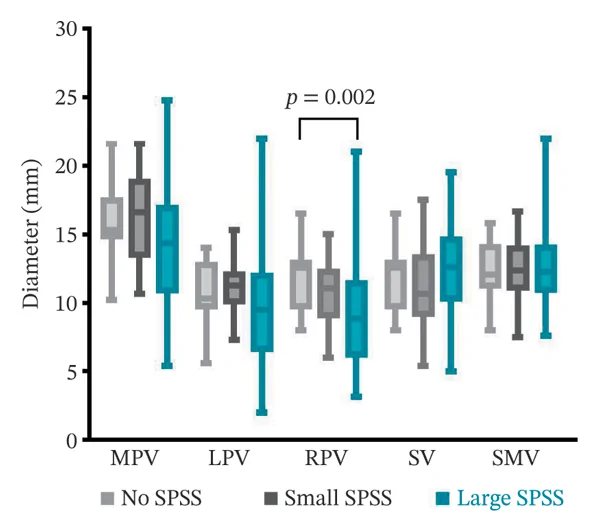
Diameters of portal vein branches across the three groups. The diameters of portal vein branches, including the main portal vein (MPV), left portal vein (LPV), right portal vein (RPV), splenic vein (SV), and superior mesenteric vein (SMV), were compared among the three groups stratified based on the status of SPSS and the diameter of the shunt tract (*φ*): no SPSS (none or *φ* < 3 mm), small SPSS (3 mm ≤ *φ* < 8 mm), and large SPSS (*φ* ≥ 8 mm). The RPV was significantly smaller in the large SPSS group compared to the no SPSS group.

Imaging measurements of the diameters of the MPV, LPV, RPV, SV, and SMV revealed that the RPV in the large SPSS group was significantly smaller than that in the no SPSS group (9.1 vs. 11.9, *p* = 0.002), whereas no significant differences were observed among the other groups (all *p* > 0.05) (Figure 1).

### 3.3. Comparison of Baseline Decompensation Events

**FIGURE 2:**
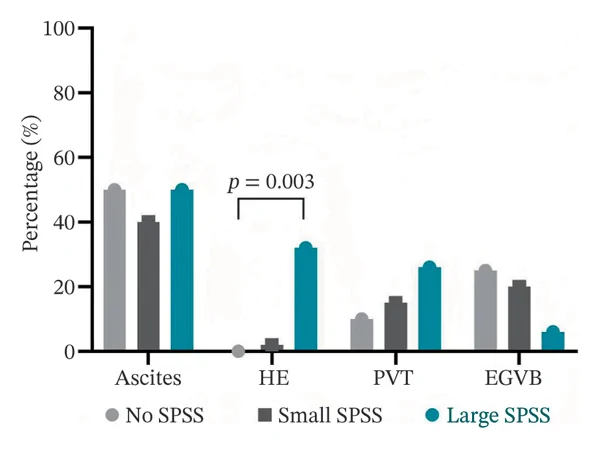
Comparison of decompensation events among the three groups. Decompensation events, including ascites, portal vein thrombosis (PVT), esophagogastric variceal bleeding (EGVB), and hepatic encephalopathy (HE), were compared across the three groups: no SPSS (none or *φ* < 3 mm), small SPSS (3 mm ≤ *φ* < 8 mm), and large SPSS (*φ* ≥ 8 mm). The incidence of HE was significantly higher in the large SPSS group compared to the no SPSS group.

Regarding baseline decompensation events (Figure 2), the incidence of ascites, PVT, and esophagogastric variceal bleeding (EGVB) was comparable across the groups (all *p* > 0.05). Notably, the incidence of HE was significantly lower in the no SPSS group compared to the large SPSS group (0% vs. 32%, *p* = 0.003).

### 3.4. Determination of SPSS Diameter Threshold

**FIGURE 3:**
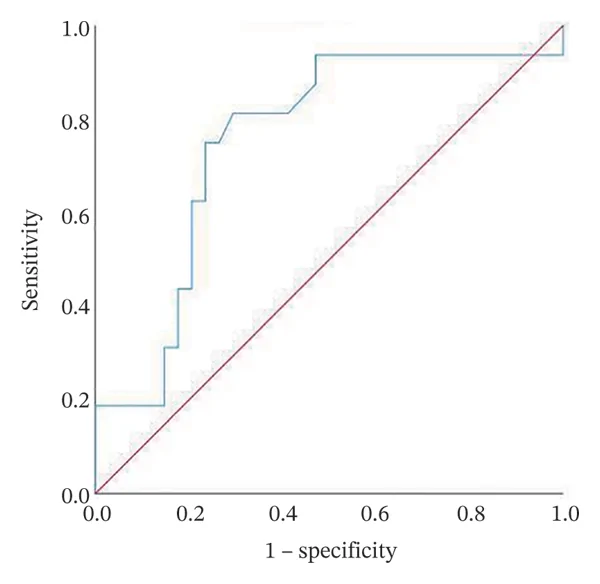
Receiver operating characteristic (ROC) curve for the determination of the optimal cutoff value for stratifying large SPSS cases into large and ultra‐large SPSS subgroups. The ROC curve was plotted using SPSS diameter as the independent variable and baseline HE occurrence as the outcome variable. The AUC was calculated with the highest value used to identify the optimal cutoff value for the shunt tract diameter.

Based on preliminary analyses showing no significant differences in HVPG values among the three groups (Table 1), further investigation was conducted to explore heterogeneity within the large SPSS group. To assess the potential impact of a wider range of SPSS diameters on HVPG values, only the decompensation event of HE showed a significant difference among the groups in the previous baseline results (Figure 2). We also evaluated other decompensation events (e.g., ascites, EGVB, and PVT) using ROC analyses. However, none of these yielded a statistically significant discriminatory threshold (all areas under the curve [AUCs] were nonsignificant). Therefore, using HE occurrence as the outcome variable and SPSS diameter as the independent variable, we stratified the large SPSS cases (*φ* ≥ 8 mm) based on an ROC‐derived cutoff value and calculated the area under the curve (AUC). The results are shown in Figure 3; the highest AUC (0.755) corresponded to an optimal shunt tract diameter cutoff of 14.5 mm, yielding a sensitivity of 81.3% and a specificity of 70.6%. Based on this cutoff value, the large SPSS cases were divided into two subgroups: large SPSS subgroup (8 mm ≤ *φ* < 14.5 mm, *n* = 27) and ultra‐large SPSS subgroup (*φ* ≥ 14.5 mm, *n* = 23). Two representative images are presented in Figure 4, showing a large SPSS (8 mm ≤ *φ* < 14.5 mm, Figure 4(a)) and an ultra‐large SPSS (*φ* ≥ 14.5 mm, Figure 4(b)).

FIGURE 4CT 3D reconstruction of portal vein images from two cases with combined large SPSS of different diameters. (a) Case in the large SPSS subgroup. (b) Case in the ultra‐large SPSS subgroup. Arrows indicate the SPSS.

**Figure:**
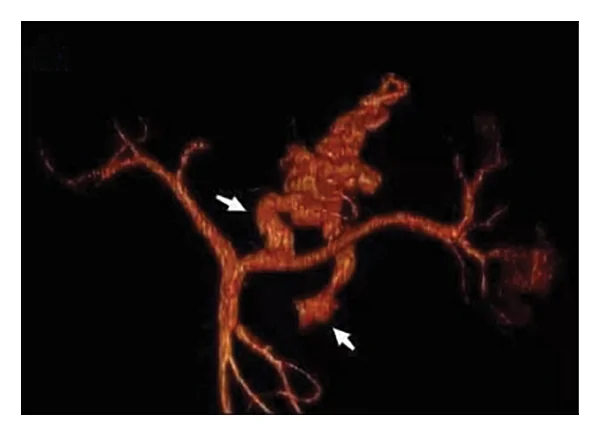
(a)

**Figure:**
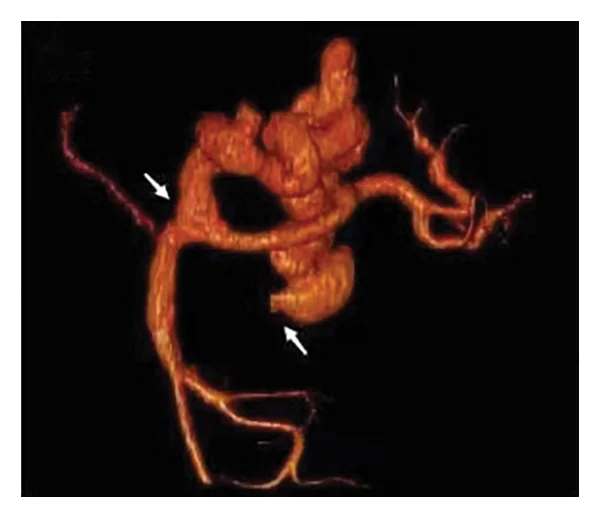
(b)

### 3.5. Clinical Characteristics of the Large SPSS Subgroup

The baseline characteristics of the two subgroups were analyzed, and the results are summarized in Table 2. There were no significant differences in gender, age, etiology, MELD‐Na, or liver/spleen volume ratio between the large SPSS and ultra‐large SPSS subgroups (all *p* > 0.05). However, significant differences were observed in the following indices: albumin: 29.6 vs. 33.1, *p* = 0.035; PT: 17.6 vs. 15.5, *p* = 0.011; INR: 1.4 vs. 1.2, *p* = 0.011; and total bilirubin: 36.8 vs. 24.8, *p* = 0.021. In terms of liver function scores, the ultra‐large SPSS subgroup had significantly worse liver function scores: Child–Pugh: 7 vs. 9, *p* = 0.005, and MELD: 14 vs. 11.6, *p* = 0.003. In addition, the ultra‐large SPSS subgroup had a sufficiently higher percentage of Grade C liver function (39.13% vs. 11%, *p* = 0.011). HVPG values were also significantly higher in the ultra‐large SPSS subgroup compared to the large SPSS subgroup (18.7 vs. 16.6, *p* = 0.03).

**TABLE 2:** Comparison of characteristics of patients in the large SPSS subgroup and ultra‐large SPSS subgroup.

	Total large SPSS cases (*n* = 50)	Large SPSS subgroup (*n* = 27)	Ultra‐large SPSS subgroup (*n* = 23)	*p* value
Gender				0.781
Female	11 (26%)	8 (30%)	6 (26.01%)	
Male	32 (74%)	19 (70%)	17 (73.91%)	
Age	53.5 ± 10	53.7 ± 10	54.9 ± 11.4	0.682
Etiology				0.827
HBV	31 (72%)	18 (67%)	16 (69.57%)	
Others	12 (28%)	9 (33%)	7 (30.43%)	
Albumin (g/L)	31.5 ± 5.9	33.1 ± 6	29.6 ± 5.5	0.035
Total bilirubin (μmol/L)	32.4 (21.5–50.8)	24.8 (19.3–41.4)	36.8 (32.3–61.9)	0.021
PT (s)	16.7 (15.3–18.6)	15.5 (14.9–17.3)	17.6 (16.1–20.2)	0.011
INR	1.3 (1.2–1.6)	1.2 (1.1–1.5)	1.4 (1.3–1.7)	0.011
Child–Pugh	8 (6–9.2)	7 (6–9)	9 (8–10)	0.005
Child–Pugh grade				0.011
A	13 (30%)	11 (41%)	2 (8.7%)	
B	19 (44%)	13 (48%)	12 (52.17%)	
C	11 (26%)	3 (11%)	9 (39.13%)	
MELD	12.9 ± 3.5	11.6 ± 2.8	14 ± 3.7	0.003
MELD‐Na	12.2 ± 4	11.7 ± 2.9	12.9 ± 5	0.292
Liver/spleen volume ratio	1.1 (0.9–1.6)	1.2 (0.9–1.8)	1 (0.9–1.3)	0.453
HVPG values	17.6 ± 3.4	16.6 ± 3.6	18.7 ± 2.89	0.030

*Note:* MELD, Model for End‐stage Liver Disease.

Abbreviations: EGVB, esophageal‐gastric variceal bleeding; HBV, hepatitis B virus; HVPG, hepatic venous pressure gradient; INR, international normalized ratio; PT, prothrombin time.

### 3.6. Portal Vein Diameter Comparison in the Large SPSS Subgroup

**FIGURE 5:**
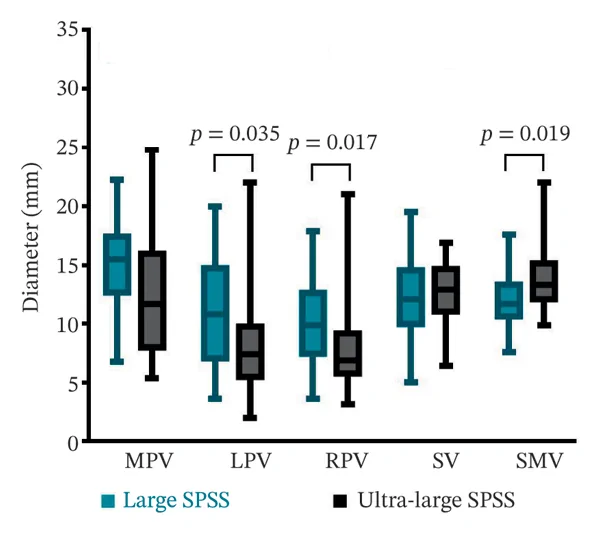
Comparison of the diameters of the portal vein branches between the two subgroups. The diameters of portal vein branches, including the main portal vein (MPV), left portal vein (LPV), right portal vein (RPV), splenic vein (SV), and superior mesenteric vein (SMV), were compared among large SPSS cases in the large SPSS subgroup and the ultra‐large SPSS group. Notably, the diameters of the LPV and RPV were smaller in the ultra‐large SPSS subgroup than in the large SPSS subgroup, whereas the diameter of the SMV was larger in the ultra‐large SPSS subgroup.

The comparison of portal vein diameters (Figure 5) revealed the following results for the ultra‐large SPSS subgroup versus the large SPSS subgroup: the diameters of the MPV (7.4 vs. 10.8, *p* = 0.035) and the RPV (6.9 vs. 9.8, *p* = 0.017) were significantly smaller in the ultra‐large subgroup, while the SMV diameter was larger (13.3 vs. 11.7, *p* = 0.019). No significant differences were observed in the diameters of the LPV and SV (*p* > 0.05).

### 3.7. Decompensation Events in the Large SPSS Subgroup

**FIGURE 6:**
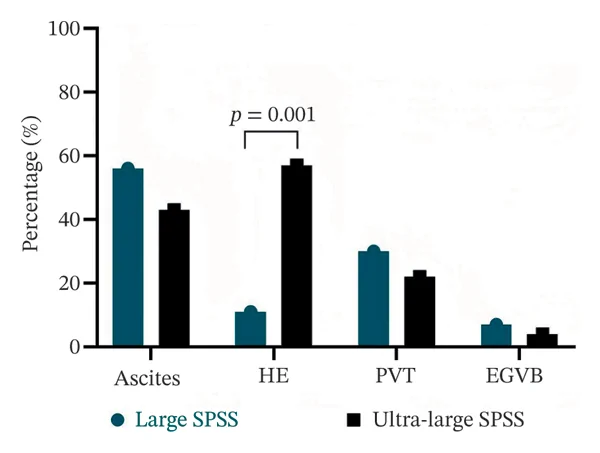
Comparison of decompensation events between the two subgroups. Decompensation events, including ascites, portal vein thrombosis (PVT), esophagogastric variceal bleeding (EGVB), and hepatic encephalopathy (HE), were compared between the large SPSS subgroup and ultra‐large SPSS subgroup. The incidence of HE was significantly higher in the ultra‐large SPSS subgroup than in the large SPSS subgroup.

The incidence of the baseline decompensation event, HE, was significantly higher in the ultra‐large SPSS subgroup than in the large SPSS subgroup (55.52% vs. 11%, *p* = 0.001). No significant differences were found in the incidence of ascites, EGVB, or PVT (*p* ≥ 0.05) (Figure 6).

### 3.8. Correlation Analysis

**FIGURE 7:**
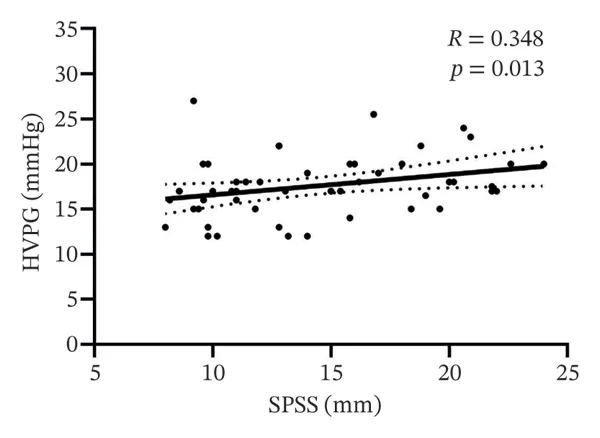
Correlation between SPSS diameter and HVPG values. Spearman’s rank correlation was conducted to examine the spontaneous portosystemic shunts (SPSSs) relationship between the diameter of the SPSS and hepatic venous pressure gradient (HVPG) values in patients with cirrhosis.

**FIGURE 8:**
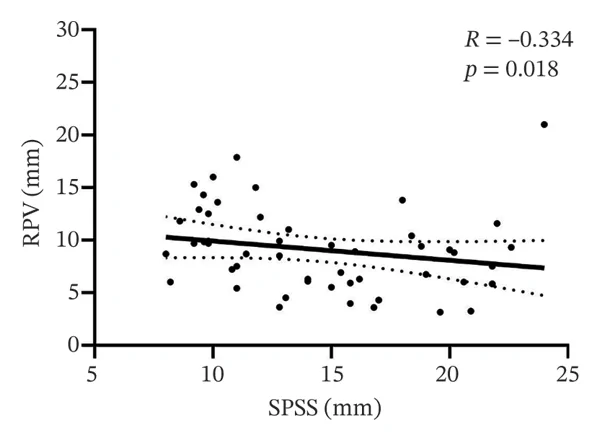
Correlation between SPSS diameter and RPV diameter. Spearman’s rank correlation was conducted to examine the relationship between the diameters of spontaneous portosystemic shunts (SPSSs) and the right portal vein (RPV) in patients with cirrhosis.

**FIGURE 9:**
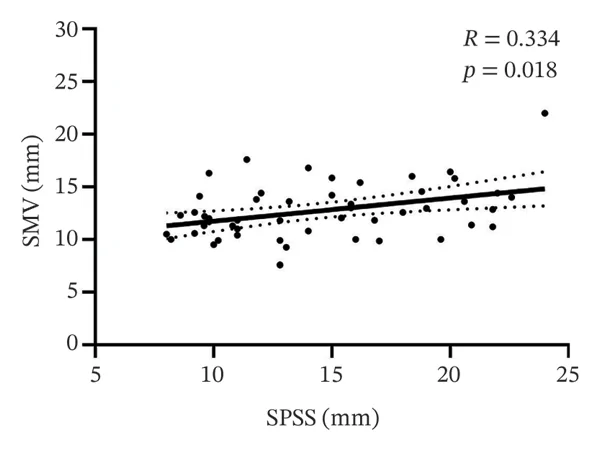
Correlation between SPSS diameter and SMV diameter. Spearman’s rank correlation was applied to assess the relationship between the diameters of the spontaneous portosystemic shunts (SPSSs) and the superior mesenteric vein (SMV) in patients with cirrhosis.

The correlation between SPSS diameter and HVPG values, intrahepatic vessel diameters, and the liver‐to‐spleen volume ratio was analyzed (Table 3). The results demonstrated a significant positive correlation between SPSS diameter and HVPG values (*R* = 0.314, *p* = 0.013), indicating that HVPG values increased with larger SPSS diameters (Figure 7). Conversely, a negative correlation was observed between SPSS diameter and RPV diameter (*R* = −0.334, *p* = 0.018) (Figure 8), indicating that larger SPSS diameters were associated with smaller RPV diameters. In contrast, a positive correlation was found between SPSS diameter and SMV diameter (*R* = 0.334, *p* = 0.018) (Figure 9), with the SMV diameter increasing alongside the SPSS diameter (Figure 9). However, no significant correlations were found between SPSS diameter and the diameters of the MPV, SV, or the liver‐to‐spleen volume ratio (all *p* > 0.05).

**TABLE 3:** Correlation of SPSS diameter with HVPG values and diameters of the MPV, LPV, RPV, SV, SMV, and liver/spleen volume ratio.

		HVPG value	MPV diameter	LPV diameter	RPV diameter	SV diameter	SMV diameter	Liver/spleen volume ratio
SPSS diameter	Spielman relevance	0.348^∗^	−0.231	−0.241	−0.334^∗^	0.135	0.334^∗^	−0.18
	Sig. (bicameral)	0.013	0.107	0.146	0.018	0.351	0.018	0.253
	Number of cases	50	50	50	50	50	50	50

^∗^A statistically significant correlation between variables.

## 4. Discussion

The impact of SPSS on patients with cirrhosis is still under debate. Recent studies have shown a higher prevalence of SPSS in cirrhotic patients, particularly those with combined SPSS, who experience more severe liver impairment and a higher incidence of complications, such as recurrent HE, gastrointestinal bleeding, ascites, and spontaneous bacterial peritonitis [7, 15, 19]. Our findings align with these previous observations, indicating that, in addition to higher total bilirubin levels, the incidence of HE in baseline decompensated events was higher in the large SPSS group compared to the no SPSS group. Moreover, the ultra‐large SPSS subgroup demonstrated worse liver function, as evidenced by significantly lower albumin, higher total bilirubin levels, prolonged PT, increased INR, and poorer liver function scores (Child–Pugh and MELD), and a higher proportion of Child−Pugh Class C patients. The incidence of HE was also significantly higher in the ultra‐large SPSS subgroup compared to the large SPSS subgroup. These findings suggest that a larger diameter SPSS may reflect a more severe state of portal hypertension and hepatic decompensation and is associated with an increased risk of decompensated events.

Although SPSS has long been recognized as a compensatory mechanism that alleviates portal pressure in portal hypertension, its hypotensive effect remains controversial. In this study, HVPG values across three groups (no SPSS, small SPSS, and large SPSS), categorized according to conventional groupings, showed no significant differences. However, further subdivision of the large SPSS group by a diameter cutoff of 14.5 mm revealed that the ultra‐large SPSS subgroup had a significantly higher HVPG value than the large SPSS subgroup. The weak‐to‐moderate correlation between SPSS diameter and HVPG (*R* = 0.314, *p* = 0.013) indicates that multiple factors influence HVPG. SPSS diameter accounts for only a limited proportion of HVPG variance, highlighting the multifactorial regulation of HVPG by variables such as PVT, splenic volume, mesenteric hemodynamics, and collateral circulation. The hemodynamic impact of SPSS may vary by anatomical location; however, this factor was not stratified in our analysis due to the retrospective design and sample size. Our findings contrast with reports suggesting HVPG decreases with larger shunts [20], a discrepancy possibly due to the inclusion of widely varying shunt sizes in other cohorts. Unlike fixed‐diameter TIPS stents (typically ≤ 8 mm) [21], the native SPSS in our study were ≥ 8 mm. We propose that an SPSS diameter exceeding 14.5 mm may signify a hemodynamic turning point. At this threshold, excessive portosystemic shunting allows neurotoxins, such as ammonia, to bypass hepatic clearance, directly promoting HE. Simultaneously, this shunting drastically reduces effective hepatic blood flow, exacerbating hepatocellular ischemic atrophy and dysfunction [22]. This progressive loss of intrahepatic vasculature and parenchymal injury increases intrahepatic resistance, further elevating HVPG. Thus, the threshold is associated with a cascade of interrelated pathophysiological processes driven by “overshunting,” rather than solely with the risk of HE. Future prospective studies with larger samples and comprehensive hemodynamic and anatomical assessments are needed to better define this critical threshold.

There are relatively few reports on the effects of SPSS on the vascularity of the portal system. The diameters of vessels within the portal vein system can reflect the blood flow and may help assess the impact of SPSS on hepatic perfusion [6, 7]. In the imaging results of this study (Figures 1 and 5) and the correlation analysis (Table 3), we observed a negative correlation trend between the diameters of the RPV and the diameter of the SPSS. Specifically, as the SPSS diameter increased, the diameters of the RPV tended to decrease. The trend suggests that larger SPSS may reduce the vascular caliber of the portal system. This reduction may reflect diminished hepatic perfusion, potentially leading to further liver function deterioration and contributing to the development of “Portal Shunt Syndrome,” characterized by intrahepatic blood vessel loss, liver atrophy, and PVT due to reduced hepatic perfusion from large SPSS.

In cirrhotic patients with preserved liver function, the presence of SPSS increases the risk of HE, variceal bleeding, and ascites and is associated with shorter survival rates [7, 21]. In the present study, we found that larger SPSS diameters were associated with a higher incidence of HE, suggesting that increased blood flow bypassing the liver hinders hepatic detoxification and contributes to HE development. HE is a leading cause of death in patients with end‐stage liver disease [23], making early recognition, removal of causative factors, and prompt treatment crucial for improving prognosis. SPSS is a recognized causative factor for recurrent HE and serves as a natural target for treatment [24–28]. The EASL guidelines recommend SPSS embolization for cirrhotic patients with recurrent or persistent HE who have failed adequate pharmacological therapy [29]. Several interventional embolization techniques have been reported for the management of SPSS, including percutaneous transhepatic embolization, balloon‐occluded retrograde transvenous obliteration, vascular plug−assisted retrograde transvenous obliteration, spring coil−assisted retrograde transvenous obliteration, and SV embolization. Some retrospective studies [30–33] have shown that patients undergoing embolization for large SPSS experience increased portal blood flow, improved liver function, a significantly reduced risk of HE recurrence, and no worsening of portal hypertension symptoms. The evidence from our study links ultra‐large SPSS (≥ 14.5 mm) to poorer outcomes, suggesting a hypothesis for future research. While current guidelines recommend SPSS embolization for drug‐refractory recurrent HE, our findings prompt the question: In patients with an ultra‐large SPSS who have not yet developed refractory HE, could preventive embolization delay liver function deterioration? This highlights the urgent need for prospective studies to evaluate the benefit‐risk ratio of embolization across different SPSS subgroups. Given the relatively small sample size and retrospective nature of existing studies, larger prospective trials are needed to confirm these observations and refine the role of shunt‐directed therapy in clinical practice.

This study has several limitations. First, the retrospective design and limited sample size preclude the exclusion of selection bias. Second, we included only patients with HVPG ≥ 10 mmHg, which may limit the generalizability of our conclusions to earlier stages of the disease. Third, this cross‐sectional analysis lacks long‐term outcome data, and follow‐up was constrained by the invasive nature of HVPG measurement and by subsequent interventional embolization in some patients. Therefore, these factors hinder the validation of the prognostic value of the 14.5‐mm cutoff. Fourth, due to the small sample size, etiological heterogeneity beyond hepatitis B could not be analyzed separately, potentially affecting the association with clinical outcomes. Future larger‐scale prospective studies with long‐term follow‐up are warranted for further exploration.

## 5. Conclusions

In conclusion, the present study suggests that a larger SPSS diameter is associated with impaired liver function and a higher incidence of HE, providing further evidence that SPSS may exacerbate hepatic dysfunction. Moreover, SPSS did not consistently lower portal pressure in this patient population. Notably, the impact of SPSS appears to diminish in a threshold‐dependent manner as the shunt diameter exceeds 15 mm, a value that may predict an increased risk of HE and liver dysfunction in cirrhotic patients. These findings support the potential utility of SPSS diameter as a clinical parameter for risk stratification and therapeutic decision‐making in the management of cirrhosis.

**Nomenclature**
SPSSs: Spontaneous portosystemic shunts
HVPG: Hepatic venous pressure gradient
HE: Hepatic encephalopathy
PT: Prothrombin time
TIPS: Transjugular intrahepatic portosystemic shunt
FHVP: Free hepatic venous pressure
WHVP: Hepatic vein wedge pressure
INR: International normalized ratio
MELD: Model for End‐Stage Liver Disease
MPV: Main portal vein
LPV: Left portal veins
RPV: Right portal veins
SMV: Superior mesenteric vein
SV: Splenic vein
PVT: Portal vein thrombosis
ROC: Receiver operating characteristic
EGVB: Esophagogastric variceal bleeding
AUC: Area under the curve

## Author Contributions

All authors contributed substantially to this study, including its conception and design. Xiao‐juan Lei and Sheng‐zhao Li performed material preparation, data collection, and analysis. Xiao‐juan Lei and Yu‐bing Jiao wrote the first draft of the manuscript. Xin‐hui Huang, Qiao Ke, and Wu‐hua Guo revised the manuscript.

## Funding

This study was supported by the Fujian Province Science and Technology Innovation Joint Funding Project (Grant no. 2021Y9033, to Wu‐hua Guo), the Fujian Natural Science Foundation (Grant nos. 2022J011286, to Yu‐bing Jiao, and 2025J011320, to Xiao‐juan Lei), and the Key Clinical Specialty Discipline Construction Program of Fuzhou, Fujian, China (Grant no. 20230102, to Wu‐hua Guo).

## Disclosure

All authors provided comments on previous versions of the manuscript. All authors read and approved the final manuscript.

## Ethics Statement

The study protocol was approved by the Ethics Committees of Mengchao Hepatobiliary Hospital of Fujian Medical University (Approval number: 2024_109_01). All procedures in this study conformed to the ethical standards of the institutional and national committees on human experimentation and complied with the Declaration of Helsinki (1975), as revised in 2008. Informed consent was obtained from all patients before being included in the study.

## Consent

Please see the Ethics Statement.

## Conflicts of Interest

The authors declare no conflicts of interest.

## Data Availability

The datasets generated in this study are included in this article and available from the corresponding author upon reasonable request.
